# Thrombocytopenia among Patients Undergoing Aortic Valve Replacement Using the Sutureless Perceval S Bioprosthesis: A Retrospective Study

**DOI:** 10.3390/jcm13041083

**Published:** 2024-02-14

**Authors:** Adrien Jayet, Henri Lu, Pierre Monney, Mario Verdugo-Marchese, Ziyad Gunga, Valentina Rancati, Zied Ltaief, Matthias Kirsch

**Affiliations:** 1 Division of Cardiovascular Surgery, Lausanne University Hospital, University of Lausanne, 1011 Lausanne, Switzerland; adrijayet@gmail.com (A.J.); mario.verdugo-marchese@chuv.ch (M.V.-M.); ziyad.gunga@chuv.ch (Z.G.); 2 Division of Cardiology, Lausanne University Hospital, University of Lausanne, 1011 Lausanne, Switzerland; henri.lu@chuv.ch (H.L.);; 3 Department of Anaesthesiology, Lausanne University Hospital, University of Lausanne, 1011 Lausanne, Switzerland; valentina.rancati@chuv.ch; 4 Adult Intensive Care Unit, Lausanne University Hospital, University of Lausanne, 1011 Lausanne, Switzerland; zied.ltaief@chuv.ch

**Keywords:** Perceval S valve, sutureless, bioprosthesis, aortic valve replacement, thrombocytopenia

## Abstract

Background: The sutureless Perceval S bioprosthesis is associated with postoperative thrombocytopenia. Our objectives were to compare the incidence, severity, and clinical implications of thrombocytopenia after aortic valve replacement (AVR) using the Perceval S or the Trifecta bioprosthesis. Methods: Patients who underwent AVR between March 2016 and August 2019 using the Perceval or Trifecta were retrospectively included. The primary endpoint was the nadir in platelet counts within 15 days after surgery. Secondary endpoints included postoperative hemolysis and inflammatory parameters, as well as clinical and echocardiographic outcomes. Results: Overall, 156 patients were included (Perceval, *n* = 103; Trifecta, *n* = 53). Preoperatively, there was no difference in platelet counts between the two groups. Postoperatively, the Perceval S bioprosthesis was associated with a greater decrease in platelet counts. The nadir was reached at Day 3 for both groups, but thrombocytopenia was more severe for the Perceval S (Perceval S vs. Trifecta, 89.2 ± 37.7 × 10^9^/L vs. 106.5 ± 34.1 × 10^9^/L, *p* = 0.01). No difference regarding lactate dehydrogenase, C-reactive protein, and white blood cells count was found. All-cause 30-day mortality rates (both valves, 2%, *p* = 0.98), hospital lengths of stay, and re-operation rates were similar. Conclusion: The Perceval S bioprosthesis was associated with more severe postoperative thrombocytopenia. This did not translate into higher short-term morbidity or mortality.

## 1. Introduction

Aortic stenosis stands as the most prevalent valvular heart disease, with a prevalence reaching 2% among individuals aged 65 or older [1]. Surgical aortic valve replacement (AVR) remains the gold standard for managing symptomatic severe AS in patients under 75 years of age and at low surgical risk [2]. During the past decade, sutureless bioprostheses have emerged as promising alternatives to traditional sutured bioprostheses in AVR procedures. These valves bear similarities to those utilized in transcatheter aortic valve implantation, with the benefit that the surgical approach allows for the removal of the native aortic valve and decalcification of the annulus, thus mitigating the risk of prosthesis–patient mismatch and paravalvular leak [3,4]. Additionally, the use of sutureless valves is associated with reduced cardiopulmonary bypass and aortic cross-clamp (ACC) durations, as well as excellent postoperative outcomes, including in a minimally invasive surgical setting [5,6,7,8].

The Perceval S (manufactured by LivaNova, London, UK), has been reported to be associated with a greater drop in postoperative platelet counts and a more frequent occurrence of severe thrombocytopenia (<50 × 10^9^/L), compared with standard sutured bioprostheses [9,10,11,12,13,14,15]. According to some of these reports, this phenomenon did not seem to have clinical consequences, either with regards to short-term mortality rate or risk of postoperative complications [11,14].

Our objectives were to: (1) compare the incidence, evolution, severity, and clinical implications of thrombocytopenia after AVR using the Perceval S or the Trifecta (St. Jude Medical, Inc., St. Paul, MN, USA) bioprosthesis; (2) assess whether thrombocytopenia could be related to increased hemolysis or a systemic inflammatory response using laboratory biomarkers.

## 2. Materials and Methods

### 2.1. Study Design and Patient Population

Our study was designed as a retrospective cohort study and conducted in the Division of Cardiac Surgery of Lausanne University Hospital (Centre hospitalier universitaire *vaudois*), a large tertiary referral center and teaching hospital in Lausanne, Switzerland. All consecutive patients aged 18 or older, who underwent isolated AVR or AVR associated with coronary artery bypass grafting (CABG) between March 2016 and August 2019 using the Perceval S or the Trifecta bioprosthesis, were included. Exclusion criteria included any surgical emergency, infective endocarditis, and combined surgeries other than AVR associated with CABG. The choice of the prosthesis was made by the operating surgeon after taking into consideration aortic valve pathology and aortic root anatomy. The Perceval S was contra-indicated in cases of aortic valve regurgitation or in cases of a ratio of sinotubular junction and aortic annulus dimension >1.4.

### 2.2. Ethical Statement

Our study was approved by the Vaud Cantonal Ethics Committee on research involving humans (CER-VD, decision 2020-00274 dated 13 January 2021). Patients’ consent was waived.

### 2.3. Perceval S Bioprosthesis Description and Surgical Approach

The Perceval S prosthesis is a bovine pericardial valve with three leaflets mounted on an expandable nitinol frame. It does not need to be surgically sutured to the aortic annulus, instead, the stent-mounted valve is compressed in a valve delivery system and deployed. The Perceval S is fixed in glutaraldehyde, detoxified with homocysteic acid, and stored in an aldehyde-free solution, reasons why rinsing is not required [15]. The valve is also covered with a thin layer of carbon film, which minimizes local inflammatory reaction and improves biocompatibility [16]. Finally, it is available in four different diameters: S (19–21 mm), M (21–23 mm), L (23–25 mm), and XL (25–27 mm).

The surgical procedure for implanting the Perceval S closely resembled that of a conventional valve replacement. Following the induction of routine general anesthesia, the surgeon proceeded with either a complete median sternotomy or a mini-sternotomy approach. Heparin was administered to achieve an activated clotting time of at least 400 s, followed by the initiation of standard cardiopulmonary bypass. Subsequently, after aortic cross-clamping and cardioplegia administration, a transverse aortotomy was performed at a slightly higher level compared to conventional valve surgery, specifically at the level of the pericardial fat fold. This adjustment aimed to preserve a segment of the ascending aorta above the prosthetic valve. The aortic valve was then excised, and the aortic annulus underwent decalcification. Given the characteristics of the Perceval S valve, complete decalcification of the aortic annulus is not necessary, but a smooth annular profile is preferable to ease coupling with the prosthesis and minimize the risk of paravalvular leak. Precise measurement of the aortic annulus diameter was then made using dedicated sizers. To ensure correct positioning and orientation of the prosthesis, 3 guiding-sutures were positioned 2 to 3 mm below the nadir of the resection line of the native valve leaflets. Each guiding-suture was then passed through a dedicated suture-loop on the inflow ring of the Perceval S valve. The bioprosthesis was then lowered and deployed into the recipient’s aortic annulus. After verifying the correct positioning of the valve, the guiding sutures were removed. Post-dilatation was performed for 30 s at a pressure of 2 to 4 atmospheres. Intra-operative trans-esophageal echocardiography was performed systematically to ensure proper valve positioning and function.

### 2.4. Endpoints

The primary endpoint was the maximum decrease in platelet counts (measured in ×10^9^/L) from baseline within 15 days after surgery. Secondary endpoints included the postoperative incidence of severe thrombocytopenia, postoperative biological variables (increase in lactate dehydrogenase (LDH), C-reactive protein (CRP), and white blood cells (WBC) count), clinical events (among which 30-day all-cause mortality, reoperation rate, and hospital lengths of stay), and echocardiographic parameters (among which left ventricular ejection fraction, aortic transvalvular maximal, and mean gradients). Complications that required reoperation included mediastinal bleeding, pericardial tamponade, or effusion. The platelet count was measured preoperatively (usually one day before surgery), and up to 15 days postoperatively. Severe thrombocytopenia was defined as a platelet count <50 × 10^9^/L. LDH (U/L), CRP (mg/L), and WBC counts (×10^9^/L) were measured preoperatively, then at one week and two weeks after surgery. Patients’ clinical characteristics (baseline) and echocardiographic parameters (baseline and 5 days after surgery) were also collected.

### 2.5. Statistical Analyses

Results were reported as number (percentage) for categorical variables and as mean ± standard deviation (SD), or as median (interquartile range (IQR)) for continuous variables, after assessment for normality via visual inspection of the distributions. Between-group bivariate comparisons were performed using Pearson’s χ2 test for categorical variables, and Student’s *t*-test or Mann–Whitney test for continuous variables. Regarding laboratory variables (platelets, LDH, CRP, and WBC), a comparison of postoperative against preoperative values was performed in each group (Perceval S, Trifecta) using the paired sample *t*-test, whenever the variable distribution was normal. Mortality rates were compared using a log-rank test. A two-sided *p*-value < 0.05 was used to define statistical significance. All analyses were carried out using the Stata software, version 16.0 (StataCorp LLC, College Station, TX, USA).

## 3. Results

### 3.1. Patients’ Baseline Clinical and Echocardiographic Characteristics

During the study period, 156 patients underwent AVR using either the Perceval S sutureless bioprosthesis (*n* = 103) or the stented Trifecta bioprosthesis (*n* = 53). Table 1 summarizes patients’ baseline clinical characteristics. Overall, patients in the Perceval S group had slightly, but significantly, higher body mass indexes (respectively, Perceval S vs. Trifecta, 28.2 ± 5.7 vs. 27.5 ± 3.9 kg/m^2^, *p* < 0.01). There was a trend toward a higher prevalence of hypercholesterolemia among patients of the Perceval S group (63% vs. 47%, *p* = 0.06). An inverse trend was observed for hypertension (80% vs. 91%, *p* = 0.08). No other difference was found regarding the patients’ comorbidities and surgical risk scores between the two groups. Table 2 presents patients’ baseline echocardiographic characteristics. No significant difference was found between the two groups, except a higher prevalence of moderate or severe preoperative aortic regurgitation in the Trifecta group (18% vs. 42%, *p* = 0.001).

### 3.2. Peri-Operative Data

Table 3 presents data regarding the peri-operative phase. There was a significantly higher proportion of patients who benefited from mini-sternotomy in the Perceval S group (respectively, Perceval S vs. Trifecta, 17% vs. 2%, *p* < 0.01). In the latter, shorter ACC and cardiopulmonary bypass durations were also observed (respectively, 44 (29–63) vs. 63 (47–78) min, *p* < 0.01, 58 (41–93) vs. 75 (58–105) min, *p* < 0.01).

### 3.3. Primary Endpoint

**Figure 1:**
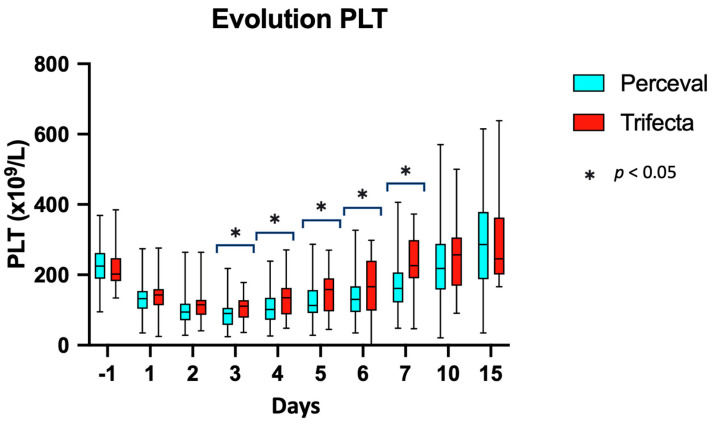
Evolution of platelet counts over time with the Perceval and Trifecta bioprostheses. PLT: platelets.

Table 4 and Figure 1 present the results. There was no significant difference regarding platelet counts at baseline between the two groups. In both cases, a postoperative decrease in platelet counts was observed from Day 1 on (statistically different from baseline values) and persisted until Day 10; the nadir of platelet counts was reached on Day 3. From Day 2 to Day 7, there was a significant difference between the platelet counts of the two groups, with those in the Perceval S group being lower (at Day 3: 89.2 ± 37.7 × 10^9^/L vs. 106.5 ± 34.1 × 10^9^/L, *p* = 0.01). Platelet counts in both groups had returned to baseline ranges by Day 10.

### 3.4. Secondary Endpoints

The incidence of severe postoperative thrombocytopenia between the Perceval S group (15%) and the Trifecta group (8%) was not statistically different (*p* = 0.30). Regarding the hemolysis and inflammatory biomarkers (LDH, CRP, and WBC count) at Days 7 and 14, there was no statistical difference either between the two groups (Table 4), although a trend toward higher LDH was found with the Perceval S at Day 7 (343 (295–406) vs. 305 (272–379) U/L, *p* = 0.09).

Table 5 presents the postoperative echocardiographic data. Patients of the Perceval S group had higher transvalvular aortic maximal and mean gradients (respectively, Perceval S vs. Trifecta, 21.8 ± 7.4 vs. 13.9 ± 5.2 mmHg, 11.9 ± 3.9 vs. 7.3 ± 2.8 mmHg), which did not translate into a statistically significant smaller effective orifice area (1.73 ± 0.52 vs. 1.84 ± 0.44 cm^2^). There was no significant difference regarding the other variables. In particular, overall, there was only one case of mild aortic paravalvular leak (Perceval S group), and no case of moderate to severe aortic paravalvular leak (*p* = 0.44).

Table 6 presents the postoperative clinical outcomes. There was no difference regarding all-cause 30-day mortality (respectively, Perceval S vs. Trifecta, 2% vs. 2%, *p* = 0.98), lengths of stay (intensive care unit: 2.47 ± 4.0 vs. 2.45 ± 4.5 days, *p* = 0.9; total: 13.2 ± 5.2 vs. 14.6 ± 13.8 days, *p* = 0.47), incidence of permanent pacemaker implantation (10% vs. 6%, *p* = 0.39), postoperative stroke (1% vs. 1.9%, *p* = 0.63), and re-operation (4% vs. 7.5%, *p* = 0.45). Postoperative atrial fibrillation was more frequent among patients who received the Trifecta valve (19% vs. 39%, *p* = 0.011).

## 4. Discussion

Our results can be summarized as follows: (1) the patients’ baseline clinical and echocardiographic characteristics between the Perceval S and Trifecta groups were similar; (2) although patients in both groups presented with a postoperative decrease in platelet counts, the use of the Perceval S bioprosthesis was associated with more profound thrombocytopenia, despite the fact that baseline platelet counts were similar. However, the incidence of severe thrombocytopenia was not different; (3) thrombocytopenia was unlikely to be related to hemolysis or an inflammatory process, given that postoperative LDH, CRP, and WBC counts were not significantly different between both groups, and did not translate into worse clinical outcomes among patients who received the Perceval S bioprosthesis.

From a biological perspective, our findings align with previously published data [10,11,12,13,14,15,17], which have documented the occurrence of thrombocytopenia associated with the Perceval bioprosthesis typically within the timeframe of 48 to 72 h after surgery. Furthermore, a gradual and complete recovery of platelet counts is commonly observed between Days 7 and 10 following the procedure. Notably, Stanger and colleagues reported a nadir in platelet counts around Day 3, with levels dropping to approximately 40% of baseline values, a pattern consistent with our own findings [12].

Regarding peri-operative details, the shorter cardiopulmonary bypass and ACC times and the higher rate of mini-sternotomy associated with sutureless bioprostheses have also been previously described [17]: this is all the more important because prolonged cardiopulmonary bypass and ACC durations have been associated with higher rates of postoperative complications [17].

From a clinical standpoint, in most studies, thrombocytopenia was not associated with worse outcomes and increased hospital lengths of stay, although some authors did report a higher need for packed blood and platelet transfusions among the patients who received the Perceval S [9,14], while Stegmeier and colleagues reported a higher rate of reoperation for bleeding (20% against 4–8%, among those who had standard bioprostheses) [15]. An interesting finding in our study is the lack of difference in the incidence of new pacemaker implantation between the two valves. This stands in contrast with previously published data [18,19]. In fact, a higher risk for high-grade atrio-ventricular block has been described in sutureless valves, which some authors attributed to balloon dilatation of the aortic annulus prior to valve implantation [17]. The reason why our data are different is unclear, but may include slightly different operative techniques, such as the lower pressure used for Perceval S post-dilatation (two atmospheres in most patients, instead of four atmospheres). Finally, the incidence of postoperative atrial fibrillation was significantly lower in the Perceval S group; to our knowledge, this observation has not been reported before. One explanation could be the shorter time of cardiopulmonary bypass in this group: cardiopulmonary bypass has indeed been shown to be an independent predictor of postoperative atrial fibrillation after CABG [20]. 

Sutureless valves do not require a suture ring, and therefore offer a larger orifice area and better postoperative hemodynamics [21]. In contrast to this, we found lower peak and mean transvalvular aortic gradients at Day 5 after surgery in the Trifecta group. This observation might be related to a higher proportion of patients in this group who were operated on for aortic regurgitation. Indeed, patients with this condition usually have a dilated aortic annulus, which allows the implantation of larger prostheses compared with patients with aortic stenosis. Of note, a recently published randomized trial, comparing sutureless against sutured AVR, did not find significantly different pressure gradients at follow-up after one year [22]. The Trifecta valve, by design (leaflets outside the frame) has shown excellent early hemodynamic results, but reports of premature structural deterioration have been published, questioning the long-term reliability of this valve [23]. 

The etiology of thrombocytopenia associated with the Perceval S bioprosthesis remains unclear and subject to ongoing debate. Various hypotheses have been proposed to explain this phenomenon. Stanger and colleagues previously posited that homocysteic acid, utilized for detoxification of the valve, might contribute by activating N-methyl-D-aspartate (NMDA) receptors in megakaryocytes (platelet precursors) and platelets, thereby initiating a cascade leading to platelet apoptosis [12]. Conversely, other researchers have suggested that the nitinol frame of the Perceval S valve could directly induce platelet membrane rupture, although the precise mechanisms behind this phenomenon remain speculative. Potential mechanisms include platelet activation, lysis by direct contact, or disruption due to turbulent flow [10,14]. Our study shows that there was no difference in postoperative increase in hemolytic or inflammatory biomarkers between the Perceval S and Trifecta groups. Thus, it appears unlikely that thrombocytopenia observed with the Perceval S valve is attributable to the platelet consumption phenomena associated with hemolytic or biological inflammatory processes. The authors, however, acknowledge that the values of LDH, CRP, and WBC during the first 6 days after surgery were not available, therefore, a correlation between the more profound drop in platelet counts associated with the Perceval S valve, and more important, abnormalities of these parameters during this timeframe, cannot be excluded. Also, a correlation between the drop in platelet counts and the postoperative higher aortic transvalvular gradients in the Perceval S group seems unlikely; in a previous study, more severe thrombocytopenia was observed despite a *lower* mean gradient among patients who received the Perceval S [9].

This study has several limitations. First, it was retrospective in design and conducted in a single center, which limits the generalizability of the results, however, it included one of the biggest numbers of patients in the Perceval S valve group compared with previously published data. Second, potential confounders that could have accounted for the decrease in platelet counts were not taken into account by adjustment analyses, however, patients at baseline did not present any significant and clinically meaningful difference regarding their clinical and echocardiographic characteristics. Third, although LDH is very sensitive to hemolysis, it also suffers from low specificity. More specific biomarkers, such as haptoglobin or schistocytes were not available. Furthermore, some important clinical postoperative bleeding complications were not recorded. Finally, a factor that could not be taken into account in the analyses is the operators’ experience in performing AVR using the Perceval S valve, and how much this could have impacted the outcomes is unknown.

## 5. Conclusions

The use of the Perceval S bioprosthesis was associated with more profound postoperative thrombocytopenia, which did not translate into worse clinical outcomes. Thrombocytopenia was unlikely to be related to hemolysis or an inflammatory process and did not translate into increased incidence of adverse clinical outcomes. Precise pathophysiological mechanisms behind this phenomenon remain unclear. Nonetheless, it is the authors’ opinion that the Perceval S valve stands out for its ease and speed of implantation and can be considered a safe device that adds to the surgeon’s therapeutic arsenal of severe aortic stenosis. Which patients would benefit the most from this device remains currently unclear. Larger and prospective clinical trials would help answer this question and are mandatory to confirm the safety and efficacy of the use of the Perceval S bioprosthesis.

## Figures and Tables

**Table 1:** Baseline demographic characteristics.

Variables	All Patients (*n* = 156)	Trifecta (*n* = 53)	Perceval (*n* = 103)	*p*-Value
Age	73.5 ± 7.7	73.9 ± 0.72	72.8 ± 1.15	0.40
Female gender	52 (33.3%)	16 (30.2%)	36 (35.0%)	0.55
Height (cm)	167.7 ± 16.3	168.5 ± 10.0	167.3 ± 9.7	0.50
Weight (kg)	78.9 ± 16.3	78.2 ± 13.4	79.2 ± 17.7	0.71
Body mass index (kg/m^2^)	28.0 ± 5.2	27.5 ± 3.9	28.2 ± 5.7	<0.01
Previous MI	24 (15%)	8(15%)	16 (16%)	0.90
Congestive heart failure	10 (7%)	5 (9%)	5 (5%)	0.28
Current smoker	18 (12%)	6 (11%)	12 (12%)	0.46
Insulin-dependent diabetes	12 (8%)	3 (6%)	9 (9%)	0.52
Hypertension	130 (83%)	48 (91%)	82 (80%)	0.08
Hypercholesterolemia	90 (58%)	25 (47%)	65 (63%)	0.06
COPD	22 (14%)	6 (11%)	16 (16%)	0.50
Cerebrovascular arteriopathy	8 (5%)	2 (4%)	6 (6%)	0.58
LEAD	34 (22%)	12 (23%)	22 (21%)	0.85
Poor mobility	8 (5%)	1 (2%)	7 (7%)	0.19
Euroscore II	1.97 (1.25–3.92)	1.92 (1.17–3.90)	1.98 (1.29–3.93)	0.98
<4%	121 (78%)	40 (76%)	81 (79%)	0.71
4 to 8%	24 (15%)	8 (15%)	16 (16%)	
>8%	11 (7%)	5 (9%)	6 (6%)	
Redo surgery	9 (6%)	4 (8%)	5 (5%)	0.50
Redo AVR	8 (5%)	3 (6%)	5 (5%)	0.83

MI: myocardial infarction, NYHA: New York Heart Association, COPD: chronic obstructive pulmonary disease, LEAD: lower extremity artery disease, AVR: aortic valve replacement.

**Table 2:** Baseline echocardiographic characteristics.

Variables	All Patients (*n* = 156)	Trifecta (*n* = 53)	Perceval S (*n* = 103)	*p*-Value
LVEF (%)	59.0 ± 11.4	57.8 ± 13.3	59.6 ± 10.4	0.42
Transvalvular aortic max gradient (mmHg)	62.2 ± 28.1	55.3 ± 29.7	65.3 ± 26.9	0.06
Transvalvular aortic mean gradient (mmHg)	38.3 ± 17.3	36.7 ± 18.1	39.0 ± 17.0	0.48
Aortic valve surface area (cm^2^)	0.79 ± 0.27	0.78 ± 0.29	0.80 ± 0.26	0.70
Bicuspid aortic valve	29 (19%)	13 (25%)	16 (16%)	0.17
Moderate or severe AR	40 (26%)	22 (42%)	18 (18%)	<0.01

LVEF: left ventricular ejection fraction, max: maximal, Vmax: maximal velocity, AR: aortic regurgitation.

**Table 3:** Peri-operative data.

Variables	All Patients (*n* = 156)	Trifecta (*n* = 53)	Perceval S (*n* = 103)	*p*-Value
Mini-sternotomy	18 (12%)	1 (2%)	17 (17%)	<0.01
CPB duration (min)	65 (48–98)	75 (58–105)	58 (41–93)	<0.01
ACC duration (min)	50 (25–73)	63 (47–78)	44 (29–63)	<0.01
Valve Size		19 mm, 2 (4%)	S, 5 (5%)	NA
		21 mm, 6 (11%)	M, 19 (18%)	
		23 mm, 12 (23%)	L, 31 (30%)	
		25 mm, 24 (45%)	XL, 48 (47%)	
		27 mm, 7 (13%)	-	
		29 mm, 2 (4%)	-	
Combined AVR/CABG	68 (44%)	22 (42%)	46 (45%)	0.71
Number of grafts	1.9 ± 0.96	2.0 ± 1.1	1.9 ± 0.91	0.74

CPB: cardiopulmonary bypass, ACC: aortic cross-clamp, AVR: aortic valve replacement, CABG: coronary artery bypass graft. NA: not applicable.

**Table 4:** Biological data.

Variables	All Patients (*n* = 156)	Trifecta (*n* = 53)	Perceval S (*n* = 103)	*p*-Value
Platelets (×10^9^/L)				
Preoperative	226.3 ± 54.5	218.8 ± 52.3	230.2 ± 55.5	0.22
Day 1	135.9 ± 42.2 *	141.3 ± 41.5 *	133.1 ± 42.4 *	0.26
Day 2	106.3 ± 41.5 *	114.0 ± 37.9 *	100.6 ± 42.8 *	0.06
Day 3	95.0 ± 37.3 *	106.5 ± 34.1 *	89.2 ± 37.7 *	0.01
Day 4	116.5 ± 51.2 *	131.2 ± 49.1 *	108.0 ± 50.7 *	0.02
Day 5	134.2 ± 58.4 *	150.5 ± 59.6 *	126.7 ± 56.7 *	0.04
Day 6	150.0 ± 72.3 *	173.8 ± 79.2 *	138.8 ± 66.6 *	0.05
Day 7	186.0 ± 85.0 *	226.0 ± 91.2	170.8 ± 77.9 *	<0.01
Day 10	238.0 ± 109.7	256.8 ± 110.4 *	229.1 ± 109.1	0.28
Day 15	288.3 ± 131.1 *	289.9 ± 126.7 *	287.1 ± 135.3	0.96
Last day	255.0 ± 117.9	276.7 ± 108.4	244.9 ± 212.4	0.16
Postoperative severe thrombocytopenia LDH (U/L)	19 (12%)	4 (8%)	15 (15%)	0.3
Preoperative	200 (171–240)	206 (173–237)	196 (170–240)	0.49
Week 1	336 (284–393)	305 (272–379)	343 (295–406)	0.09
Week 2	423 (339–502)	543 (364–635)	405 (339–463)	0.33
CRP (mg/L)				
Preoperative	9.2 ± 33.0	13.2 ± 51.3	7.0 ± 15.8	0.39
Week 1	144.2 ± 68.7 *	145.4 ± 68.2 *	143.5 ± 69.3 *	0.87
Week 2	58.9 ± 50.1 *	64.9 ± 55.8 *	56.1 ± 47.4 *	0.45
WBC (×10^9^/L)				
Preoperative	7.8 ± 2.6	7.4 ± 1.9	8.1 ± 2.9	0.11
Week 1	12.7 ± 4.1 *	13.0 ± 4.2 *	12.5 ± 4.0 *	0.44
Week 2	10.1 ± 3.9 *	9.5 ± 2.6	10.4 ± 4.3 *	0.25

* *p* < 0.01 vs. preoperative values, using the paired sample *t*-test. LDH: lactate dehydrogenase, CRP: C-reactive protein, WBC: white blood cells.

**Table 5:** Postoperative echocardiographic data.

Variables	All Patients (*n* = 156)	Trifecta (*n* = 53)	Perceval S (*n* = 103)	*p*-Value
LVEF (%)	58.4 ± 11.3	56.1 ± 12.7	59.6 ± 10.4	0.07
Transvalvular aortic max gradient (mmHg)	19.1 ± 7.7	13.9 ± 5.2	21.8 ± 7.4	<0.01
Transvalvular aortic mean gradient (mmHg)	10.4 ± 4.2	7.3 ± 2.8	11.9 ± 3.9	<0.01
EOA (cm^2^)	1.76 ± 0.50	1.84 ± 0.44	1.73 ± 0.52	0.27
Aortic PVL				0.44
- Moderate to severe	0 (0%)	0 (0%)	0 (0%)	
- Mild	1 (3%)	0 (0%)	1 (0.7%)	

LVEF: left ventricular ejection fraction, EOA: effective orifice area, PVL: para-valvular leak.

**Table 6:** Postoperative outcomes.

Variables	All Patients (*n* = 156)	Trifecta (*n* = 53)	Perceval S (*n* = 103)	*p*-Value
30-day all-cause mortality	3 (2%)	1 (2%)	2 (2%)	0.98
Hospital length of stay (days)	13.7 ± 9.1	14.6 ± 13.8	13.2 ± 5.2	0.47
ICU length of stay (days)	2.5 ± 4.3	2.5 ± 4.5	2.5 ± 4.0	0.90
Postoperative atrial fibrillation	40 (26%)	20 (39%)	20 (19%)	0.01
New pacemaker implantation	13 (8%)	3 (6%)	10 (10%)	0.39
Postoperative stroke	2 (1.3%)	1 (1.9%)	1 (1%)	0.63
Reoperation	8 (5%)	4 (7.5%)	4 (4%)	0.45

ICU: intensive care unit.

## Data Availability

The data presented in this study are available upon reasonable request from the corresponding author.
